# Functional Brain Reorganization After Radio Electric Asymmetric Conveyer (REAC) Brain Wave Optimization Gamma (BWO-G) Neuromodulation in Individuals With Chronic Stress Exposure: A Retrospective Case Series With Multimodal Evaluation

**DOI:** 10.7759/cureus.90951

**Published:** 2025-08-25

**Authors:** Salvatore Rinaldi, Acary S Oliveira, Valeria Modestto, Arianna Rinaldi, Vania Fontani

**Affiliations:** 1 Research Department, Rinaldi Fontani Foundation, Florence, ITA; 2 Department of Regenerative Medicine, Rinaldi Fontani Institute, Florence, ITA; 3 Neurology, São Paulo Federal University, São Paulo, BRA; 4 Department of Adaptive Neuro Psycho Physio Pathology and Neuro Psycho Physical Optimization, Rinaldi Fontani Institute, Florence, ITA

**Keywords:** bioelectrical activity, chronic stress, gamma waves, ica, neuromodulation, neuroplasticity, qeeg, reac bwo-g, sloreta

## Abstract

Chronic stress exposure is a widespread and disabling condition that disrupts neurophysiological regulation and affects emotional, cognitive, and somatic domains. In this series, the term “chronic stress exposure” refers to prolonged exposure to psychosocial and occupational stressors that, although not classified as a distinct disorder in the Diagnostic and Statistical Manual of Mental Disorders, 5th Edition (DSM-5) or International Classification of Diseases, 10th Revision (ICD-10), can induce persistent dysregulation of brain networks involved in emotion and cognition. Radio Electric Asymmetric Conveyer (REAC) Brain Wave Optimization Gamma (BWO-G) is a noninvasive neuromodulation protocol aimed at restoring neural coherence by enhancing subcortical-cortical connectivity.

This retrospective case series presents five individuals with exposure to chronic stress who underwent 18 sessions of REAC BWO-G. Pre- and post-treatment evaluations included quantitative electroencephalography (qEEG), Independent Component Analysis (ICA), and standardized low-resolution brain electromagnetic tomography (sLORETA). Across all cases, post-treatment assessments revealed consistent trends of increased power symmetry in delta, theta, and alpha bands, along with frequency shifts and reorganization of dominant cortical activity toward areas associated with emotional regulation and the default mode network. Clinically, all patients reported improvements in emotional stability, sleep quality, and cognitive clarity. These findings support the potential of REAC BWO-G to modulate dysfunctional cortical dynamics associated with chronic stress exposure and highlight the utility of qEEG-based neuroimaging in monitoring therapeutic effects.

## Introduction

Chronic stress exposure is defined as prolonged exposure to persistent psychosocial and environmental stressors that induce sustained alterations in brain network activity, understood as the coordinated patterns of communication and functional connectivity among distributed brain regions that support cognitive, emotional, and physiological regulation, without necessarily meeting criteria for a psychiatric disorder. It is a pervasive and increasingly recognized public health concern [1] and is associated with cardiovascular diseases [2], metabolic syndrome [3], depression [4], anxiety [5], and neurodegenerative conditions [6]. It exerts a profound impact on brain structure [7,8] and function [9], particularly on networks involved in emotional regulation [10], executive functioning [11], and interoception [12].

Chronic stress exposure induces maladaptive neuroplastic changes, including functional connectivity disruptions within the default mode network (DMN), which is primarily involved in self-referential processing and emotion regulation [13], salience network (SN), which integrates interoceptive and emotional information and guides attentional shifts toward relevant stimuli [14], and central executive network (CEN), which supports working memory, cognitive control, and goal-directed behavior. These alterations are accompanied by persistent dysregulation of the hypothalamic-pituitary-adrenal (HPA) axis [15] and autonomic nervous system [16]. Neuroimaging and electrophysiological studies have documented these alterations, revealing patterns of increased low-frequency activity, reduced beta coherence, and altered source localization, often associated with impaired cognitive and emotional processing [17].

Recent years have witnessed a growing interest in neuromodulatory approaches capable of addressing these neurophysiological disruptions. Noninvasive neuromodulation techniques, such as transcranial magnetic stimulation (TMS) and transcranial direct current stimulation (tDCS), have shown potential but often require complex equipment and have limitations in reproducibility and accessibility [18]. The Radio Electric Asymmetric Conveyer (REAC) technology offers a distinct approach, utilizing asymmetrically conveyed radio electric fields to promote bioelectrical optimization without the need for invasive procedures or complex parameter settings [19]. Among REAC protocols, Brain Wave Optimization Gamma (BWO-G) is specifically designed to promote cortical coherence and functional reorganization by targeting the integration of subcortical and cortical circuits involved in stress regulation [20,21].

This case series investigates the neurophysiological effects of REAC BWO-G in five individuals presenting with chronic stress exposure-related dysregulation. All patients shared a common occupational environment characterized by sustained psychological pressure, interpersonal tension, and limited autonomy, to which they had been exposed for periods ranging from five to more than 10 years. Using quantitative electroencephalography (qEEG) [22], Independent Component Analysis (ICA) [23], and standardized low-resolution brain electromagnetic tomography (sLORETA) [24], this study aims to document the changes in cortical activity patterns before and after a cycle of REAC BWO-G sessions, correlating neurophysiological reorganization with reported clinical improvements.

## Case presentation

Materials and methods

The theoretical basis of REAC BWO-G lies in its ability to interact with areas of altered endogenous bioelectrical activity in target tissues through asymmetrically conveyed radioelectric fields. This interaction promotes the progressive optimization of altered bioelectrical activity induced by epigenetic processes, fostering the recovery of functional neurophysiological patterns. The specific emission parameters, including field intensity, frequency modulation, and asymmetric conveyance, are pre-set, proprietary to the REAC system, and cannot be modified by the operator. This design ensures standardized delivery and reproducibility of treatment across different patients.

The REAC BWO-G protocol is part of the family of Neuro Psycho Physical Optimization-Cervico Brachial (NPPO-CB) protocols [25]. The term Brain Wave Optimization (BWO) refers to the therapeutic focus of these protocols, which aim to restore the balanced generation of endogenous brain rhythms, including delta, theta, alpha, beta, and gamma waves when their physiological production is altered or imbalanced. Delta waves (0.5-4 Hz) are typically linked to deep sleep and restorative processes; theta waves (4-8 Hz) are associated with drowsiness, memory encoding, and emotional reactivity; alpha waves (8-12 Hz) reflect relaxed wakefulness and sensory inhibition; beta waves (13-30 Hz) are related to alertness, active cognition, and executive control; and gamma waves (>30 Hz) are essential for higher-order integrative functions, including attention, memory, and consciousness. [26]. Specifically, the BWO-G protocol is designed to facilitate the recovery of optimal gamma wave activity, which is associated with higher-order cognitive, emotional, and integrative functions that may be impaired in chronic stress and other dysregulated states.

The REAC technology employs a medical device (BENE Mod 110, ASMED, Scandicci, Italy) capable of generating low-intensity, asymmetrically conveyed radioelectric fields. Unlike conventional electrical stimulation or electromagnetic field techniques, REAC does not deliver currents or pulses directly into tissues. Instead, it interacts with the endogenous bioelectrical activity of the target areas, promoting their progressive normalization. The emission parameters (intensity, waveform, and duration) are fixed by the manufacturer (ASMED) and cannot be adjusted by the operator, ensuring consistency across treatment sessions. The emission is continuous, noninvasive, and administered through asymmetric conveyor probes (ACPs) placed bilaterally over the cervico-brachial area. This strategic positioning is intended to engage central regulatory circuits and promote top-down modulation of subcortical and cortical structures, potentially influencing vagal pathways, brainstem nuclei, and cortical areas involved in emotional and cognitive regulation.

Each patient underwent 18 sessions of REAC BWO-G neuromodulation [20,21,27], with each session lasting approximately five minutes. Sessions were separated by a minimum interval of one hour. No pharmacological or psychotherapeutic interventions were administered during the treatment period. None of the subjects reported adverse effects or perceived the treatment during or after the REAC BWO-G sessions. Follow-up clinical assessments were conducted approximately one month after the final session. Patients did not report any significant external life changes or additional therapeutic interventions during the observation period. No adverse effects were reported during or after the treatment sessions, supporting the safety and tolerability of the procedure. This methodological approach aims to standardize treatment delivery, ensure reproducibility, and facilitate objective evaluation of neurophysiological and clinical outcomes. During the REAC BWO-G sessions, patients reported no subjective perception of the treatment, in accordance with the noninvasive nature of the procedure.

This case series involved a retrospective analysis of anonymized clinical and neurophysiological data. All patients were clinically followed up for at least one month after completing the treatment cycle, during which no adverse effects were observed. Although no further follow-up was performed in this retrospective series, future prospective studies are planned to further monitor the long-term stability of clinical and neurophysiological effects and to rule out any potential harmful biological consequences. It should also be noted that the BWO protocols, formally named Neuro Psycho Physical Optimization - Cervico Brachial Brain Wave Optimization (NPPO-CB BWO), have been widely applied and investigated for many years, and no adverse or undesirable effects have ever been reported in the scientific literature or in clinical practice..

EEG recordings were acquired using a Mitsar 21-channel EEG amplifier and analyzed using WinEEG and sLORETA software. Power spectral maps were generated to evaluate frequency band distributions across cortical regions, offering a clear visual representation of brain activity before and after treatment. EEG data were visually inspected, and segments contaminated by ocular or muscle artifacts were excluded. ICA [23] was performed, extracting 21 components corresponding to the 21 EEG channels. Components identified as artifacts based on morphology, scalp distribution, and frequency content (e.g., eye blinks, muscle activity, line noise) were rejected, and only artifact-free components were retained for further analysis. Pre- and post-treatment qEEG assessments were typically conducted between 9:00 a.m. and 12:00 p.m., depending on participant availability. While efforts were made to maintain consistency, some variation in timing occurred among participants. The sLORETA analysis [24] applied a Z-score threshold >2.5 to identify areas of cortical hyperactivity before treatment. Post-treatment normalization was defined by Z-scores <1.5, following established neuroimaging standards for clinically meaningful change.

The REAC BWO-G protocol was delivered using the BENE Mod. 110 medical device (ASMED). A key feature of this technology is its lack of operator-dependent parameters. The emission is pre-programmed, continuous, and conveyed via low-intensity radioelectric fields asymmetrically. The field intensity is extremely low, comparable to everyday environmental radioelectric exposure, and the emission is not directly focused on the patient but dispersed into the surrounding environment. These features ensure both safety and tolerability, as confirmed by the absence of adverse or undesirable effects reported in this case series and in prior studies using the same protocols.

Clinical outcomes were based on patient self-reports and systematically verified during follow-up visits conducted by physicians involved in their care. No standardized psychometric scales were employed in this retrospective case series. However, previous REAC studies using other neuromodulation protocols have employed validated psychometric instruments to demonstrate significant clinical benefits.

Case presentations

All patients shared a common occupational environment, characterized by sustained psychological pressure, frequent interpersonal conflicts, strict productivity demands, and limited autonomy in decision-making. They had been exposed to this setting for periods ranging from five to more than 10 years, which contributed to the persistence of stress-related dysregulation. Despite their differing clinical manifestations and comorbidities, their symptomatology overlapped: persistent emotional dysregulation, cognitive inefficiency, sleep disturbances, musculoskeletal tension, and fatigue. Several had previously attempted pharmacological or behavioral interventions such as anxiolytics, antidepressants, dietary changes, relaxation techniques, or psychotherapy with limited or no benefit. No external life changes or interventions were reported during the observation period, supporting the attribution of clinical and neurophysiological changes to the REAC BWO-G intervention.

Case 1

A 61-year-old female presented with a complex symptomatology that had developed progressively over more than five years, characterized by persistent musculoskeletal tension and non-specific pain symptoms, emotional lability, sleep disturbances, poor concentration, and a general sense of psychophysiological malaise. She had no psychiatric history and had never undergone pharmacological or psychotherapeutic interventions. She reported resorting occasionally to over-the-counter sleep aids and anxiolytics without sustained benefit. The REAC BWO-G treatment was initiated to address these multidimensional stress-related disturbances.

**Figure 1:**
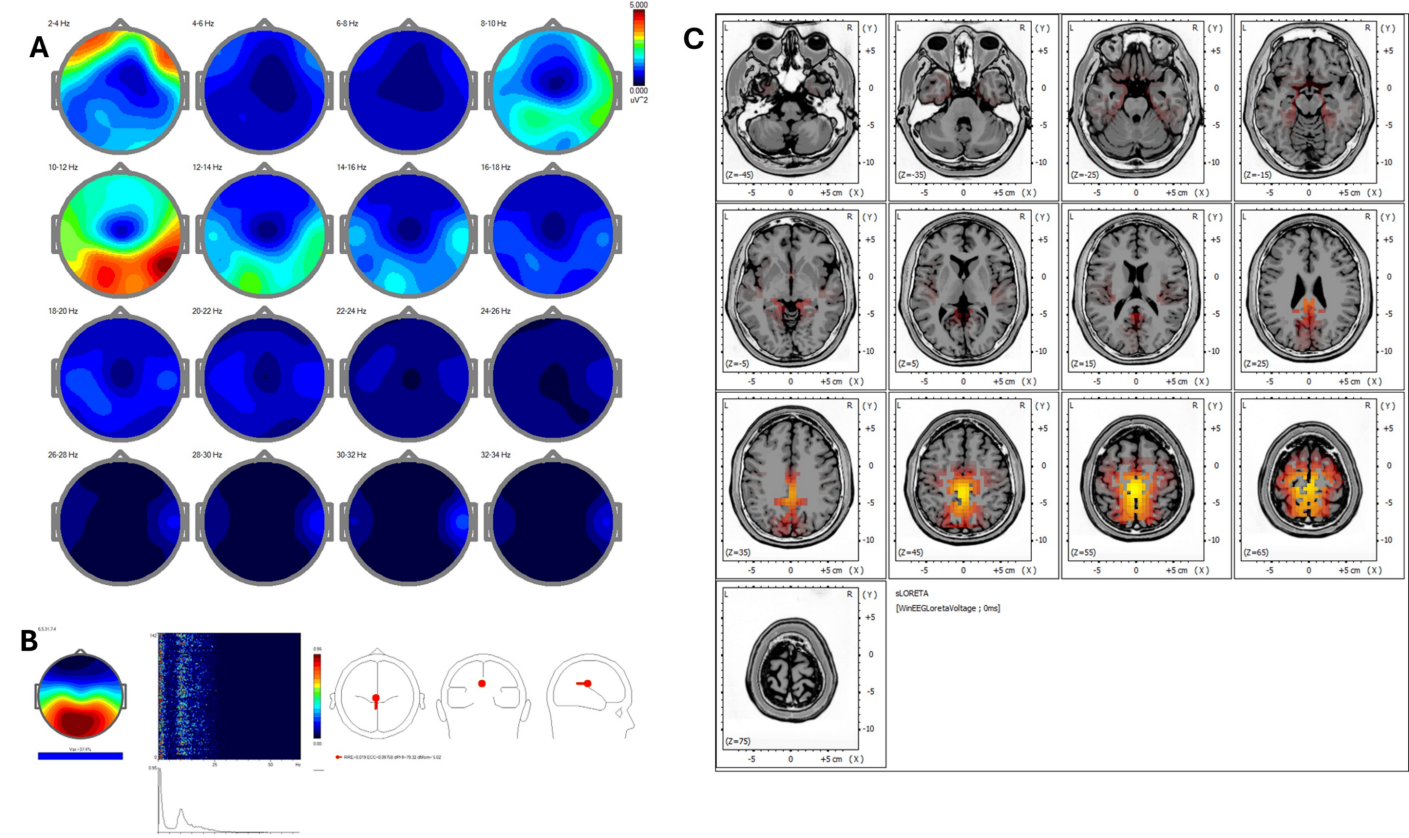
Case 1 - pre-treatment (A) EEG power spectral maps before REAC BWO-G treatment. Topographical maps of EEG power spectral density across 2-Hz frequency bands (2-34 Hz) in eyes-closed resting-state condition before the REAC BWO-G protocol. Increased power is observed predominantly in the delta (2-4 Hz) and alpha (10-12 Hz) bands, with spatial distribution focused in frontal and posterior regions, reflecting baseline cortical activity with reduced synchronization in higher frequency ranges. (B) ICA before REAC BWO-G treatment. ICA of EEG data before REAC BWO-G treatment reveals dominant frequency peaks at 0.98 Hz, 1.95 Hz, 8.79 Hz, 10.25 Hz, 12.70 Hz, and 21.23 Hz, with cortical sources localized to Brodmann areas 4, 5, 6, 7, and 31. These areas are primarily involved in sensorimotor, visuo-spatial, and premotor functions, indicating a reactive neurophysiological profile. (C) sLORETA cortical source localization before REAC BWO-G treatment. sLORETA of resting-state EEG reveals predominant activation in Brodmann areas 4, 5, 6, 7, and 31. These areas are primarily associated with primary motor and premotor functions, somatosensory integration, and visuo-spatial processing. The spatial distribution of activation suggests a cortical pattern consistent with heightened sensorimotor reactivity and environmental vigilance, often associated with stress-related functional states EEG: electroencephalography; ICA: Independent Component Analysis; REAC BWO-G: Radio Electric Asymmetric Conveyer Brain Wave Optimization Gamma; sLORETA: standardized low-resolution electromagnetic tomography

Baseline qEEG (Figure 1A) displayed increased power in delta and alpha bands predominantly in frontal and posterior cortical regions. The corresponding ICA (Figure 1B) indicated peaks at 0.98, 1.95, 8.79, 10.25, 12.70, and 21.23 Hz with activity in BA 4, 5, 6, 7, and 31, consistent with a sensorimotor-reactive pattern. Pre-treatment sLORETA (Figure 1C) highlighted hyperactivity (Z-score >2.5) in primary sensorimotor areas, specifically BA 4 and BA 6, and posterior associative areas including BA 7 and BA 31.

**Figure 2:**
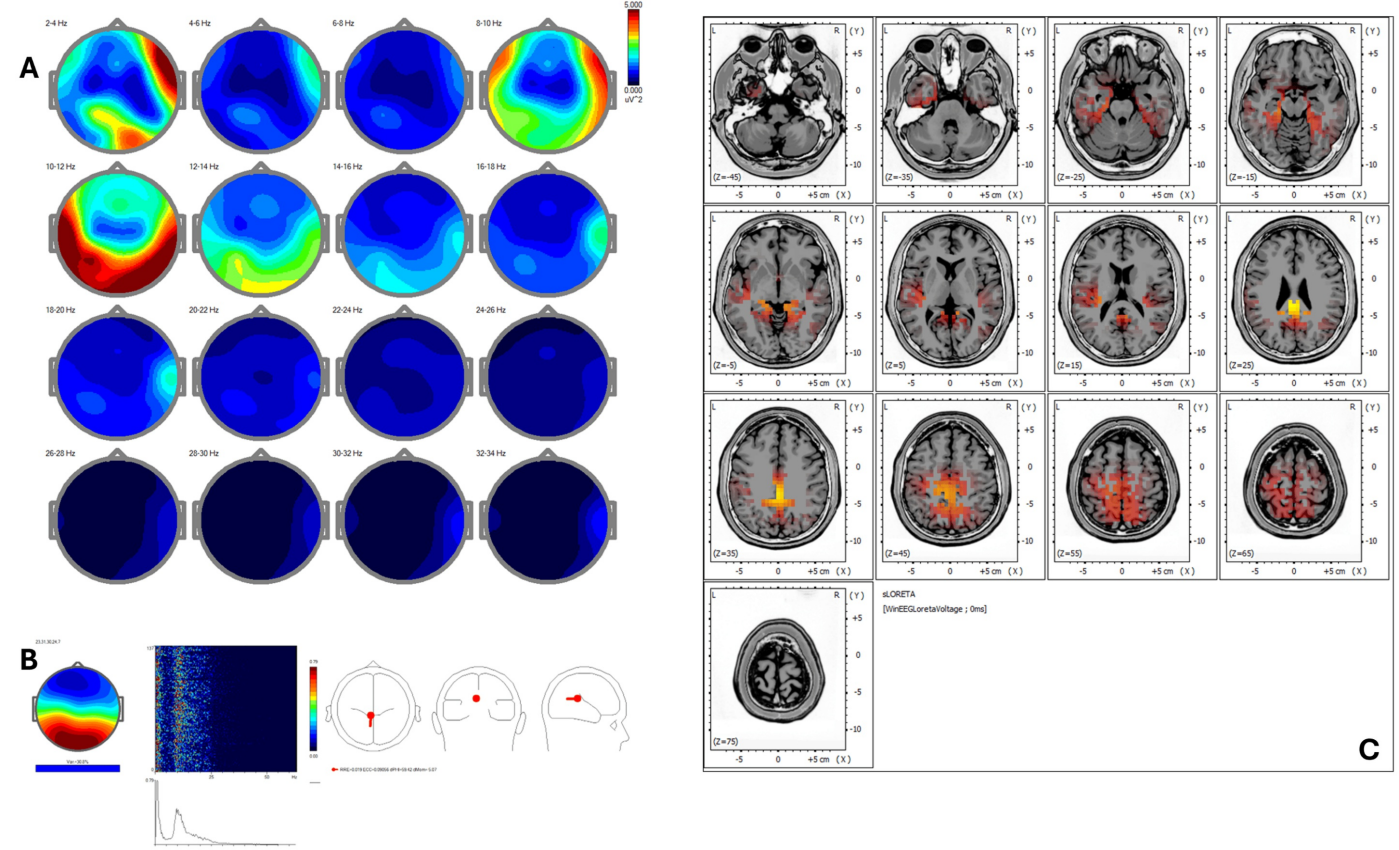
Case 1 - post-treatment (A) EEG power spectral maps after REAC BWO-G treatment. Following the complete cycle of 18 REAC BWO-G sessions, EEG power spectral maps show increased and more symmetrical power in the delta, theta, and alpha bands, with broader spatial distribution, especially in posterior areas. These changes suggest enhanced cortical synchronization and neurophysiological reorganization toward a more functionally integrated resting-state pattern. (B) ICA. Post-treatment ICA shows preserved low-frequency peaks and a shift in beta activity toward 24.17 Hz. Cortical source activity is now concentrated in Brodmann areas 23, 24, 30, 31, and 7, indicating greater involvement of the default mode and limbic-paralimbic networks. This reorganization suggests a neurophysiological shift toward improved emotional regulation and self-referential processing following REAC BWO-G treatment. (C) sLORETA cortical source localization. Post-treatment sLORETA analysis shows a redistribution of cortical activation toward Brodmann areas 23, 24, 30, 31, and 7, involving key regions of the posterior and anterior cingulate cortices and the retrosplenial cortex. These areas are functionally implicated in emotional regulation, interoceptive awareness, and self-referential cognitive processing. This case illustrates a functional reorganization from sensorimotor-dominant cortical activity to a more integrated and symmetrical midline network, aligning with the patient’s clinical recovery and the expected neuromodulatory effects of the REAC BWO-G protocol. Note: only the most prominent ICA peaks are illustrated in the figures. Minor or overlapping components described in the text may not be clearly visible due to figure resolution or graphical scaling limitations EEG: electroencephalography; ICA: Independent Component Analysis; REAC BWO-G: Radio Electric Asymmetric Conveyer Brain Wave Optimization Gamma; sLORETA: standardized low-resolution electromagnetic tomography

Post-treatment qEEG (Figure 2A) revealed an increase in coherence and spatial symmetry, while ICA (Figure 2C) showed new peaks at 9.28, 10.25, 12.70, and 24.17 Hz with activity shifting toward BA 23, 24, 30, 31, and 7, corresponding to areas involved in emotional regulation and self-referential processing. Post-treatment sLORETA (Figure 2C) demonstrated a redistribution of cortical activity to midline regions, including the posterior cingulate cortex (BA 31) and retrosplenial cortex (BA 30), with normalization of Z-scores (<1.5) in previously hyperactive areas.

Clinically, the patient reported significant improvements in sleep quality, emotional regulation, reduced musculoskeletal tension and non-specific pain symptoms, and enhanced cognitive fluidity. These improvements were confirmed during a follow-up evaluation one month after the end of the treatment cycle. No further follow-up was available in this retrospective case, although prospective studies are planned to assess the stability of effects over extended periods. No changes in external life circumstances or other therapeutic interventions were reported during this period.

Case 2

A 35-year-old male, employed in the same occupational setting, presented with chronic musculoskeletal pain, frequent headaches, facial flushing associated with rosacea, chronic fatigue, non-restorative sleep, anticipatory anxiety, and difficulty regulating eating behaviors under stress. Despite these persistent issues, he described himself as “in good health” until symptoms began to interfere with his professional performance and social interactions. Previous attempts to manage his condition through dietary adjustments, mindfulness techniques, and occasional over-the-counter medications had provided only transient and inconsistent relief.

**Figure 3:**
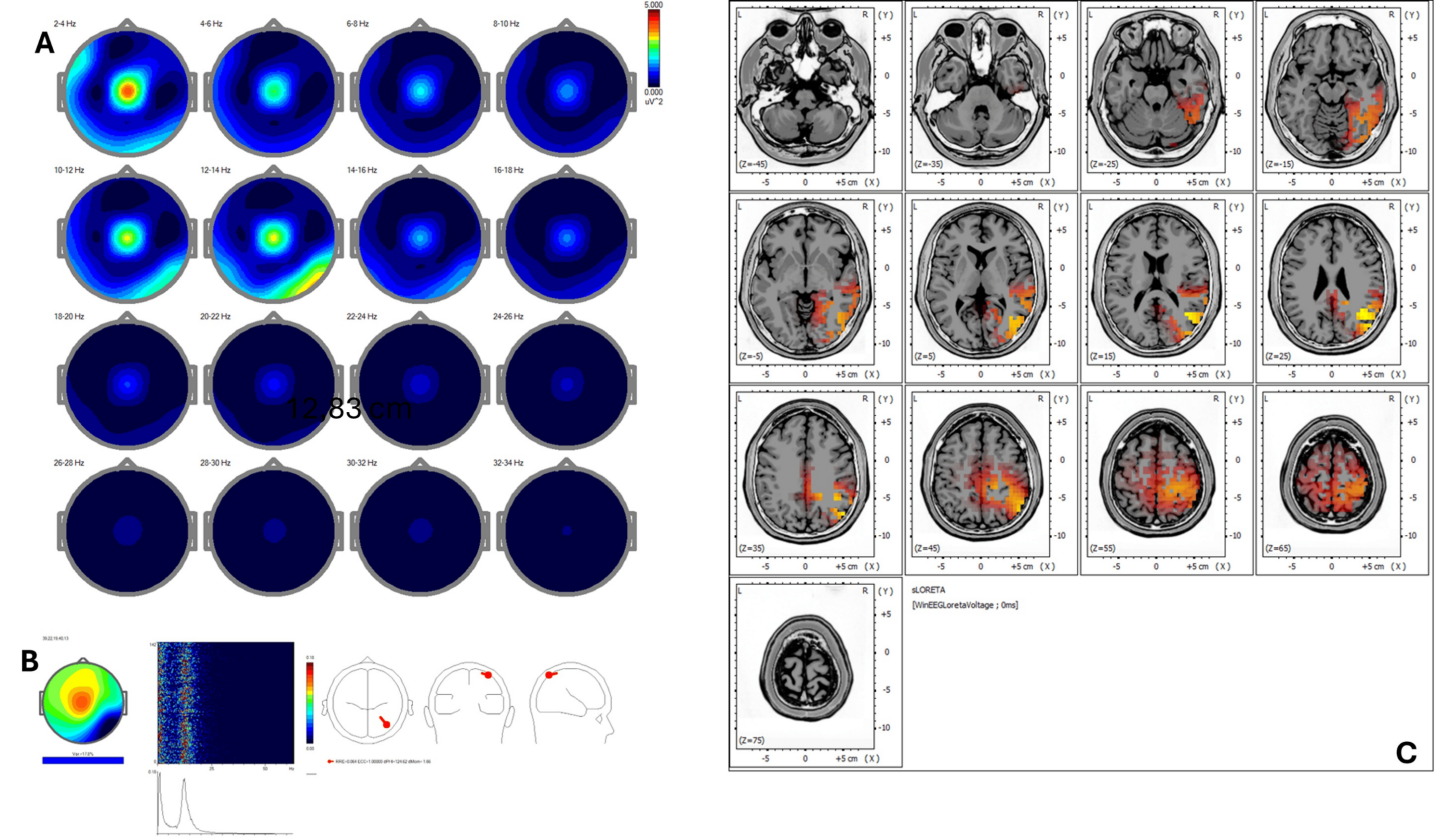
Case 2 - pre-treatment (A) EEG power spectral maps before REAC BWO-G treatment. Topographical distribution of EEG power across 2-Hz frequency bands (2-34 Hz) in eyes-closed resting state before REAC BWO-G. Elevated delta and alpha power predominates in parietotemporal regions, with reduced beta synchronization, reflecting a reactive cortical overload pattern associated with chronic stress. (B) ICA before REAC BWO-G treatment. ICA decomposition reveals frequency peaks at 1.22 Hz, 10.99 Hz, 14.89 Hz, and 17.33 Hz, with source localization in Brodmann areas 39, 22, 19, 40, and 13. These areas are implicated in sensory processing, language, and self-referential functions, suggesting a neurophysiological state of hypervigilance and cognitive dysregulation. (C) sLORETA cortical source localization before REAC BWO-G treatment. sLORETA analysis shows hyperactivity (Z-score >2.5) in the right temporoparietal junction (Brodmann areas 39 and 22), a region associated with somatic amplification and rumination, reinforcing the clinical picture of stress-related maladaptation EEG: electroencephalography; ICA: Independent Component Analysis; REAC BWO-G: Radio Electric Asymmetric Conveyer Brain Wave Optimization Gamma; sLORETA: standardized low-resolution electromagnetic tomography

Baseline qEEG revealed increased delta and theta power over frontal regions (BA 6, 8), with reduced beta coherence in parietal areas (BA 7, 40) (Figure 3A). ICA showed peaks at 1.22 Hz, 10.99 Hz, 14.89 Hz, and 17.33 Hz, with sources in BA 39, 22, 19, 40, and 13 (Figure 3B). Pre-treatment sLORETA (Figure 3C) showed hyperactivity (Z-score >2.5) in the right temporoparietal junction (BA 39, 22), consistent with somatic amplification and ruminative processing.

**Figure 4:**
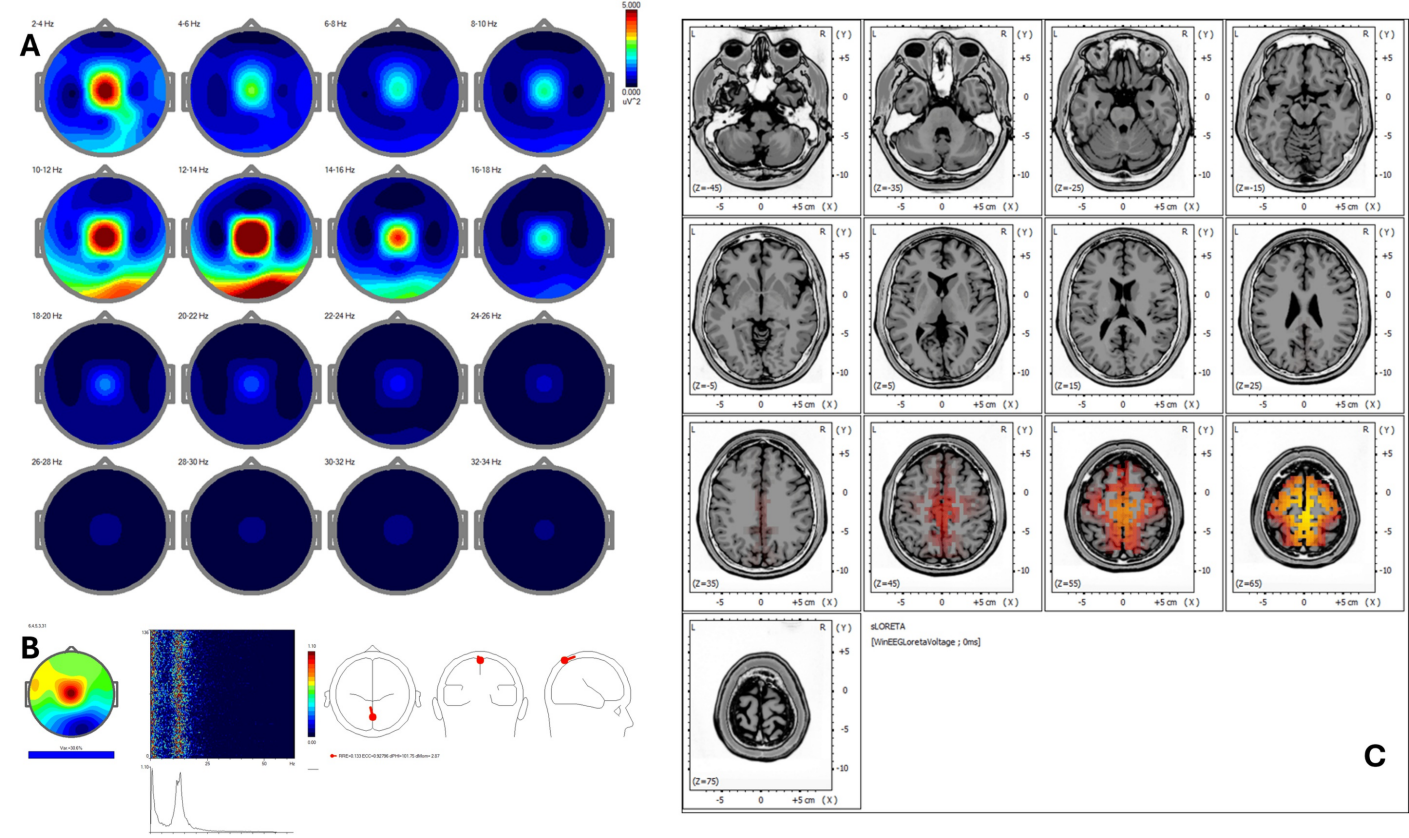
Case 2 - post-treatment (A) EEG power spectral maps after REAC BWO-G treatment. Post-treatment maps demonstrate marked improvement in spatial power symmetry, with reduction of excess delta and theta activity in frontal regions and normalization of alpha distribution in parietotemporal areas, indicating decreased hypervigilance and enhanced cortical integration. (B) ICA after REAC BWO-G treatment. ICA shows reorganized spectral peaks at 1.71 Hz, 11.47 Hz, 13.43 Hz, and 15.87 Hz, with cortical sources now redistributed toward Brodmann areas 6, 4, 5, 3, and 31. This reflects greater engagement of sensorimotor and midline networks supporting emotional regulation and attentional stability. (C) sLORETA cortical source localization after REAC BWO-G treatment. sLORETA analysis reveals resolution of hyperactivity in the right temporoparietal junction and new balanced activation in midline frontal structures (BA 24 and 31), consistent with restoration of self-regulatory and integrative cortical functions EEG: electroencephalography; ICA: Independent Component Analysis; REAC BWO-G: Radio Electric Asymmetric Conveyer Brain Wave Optimization Gamma; sLORETA: standardized low-resolution electromagnetic tomography

Post-treatment qEEG (Figure 4A) demonstrated improved power symmetry and reduced low-frequency hyperactivity. ICA shows reorganized peaks at 1.71 Hz, 11.47 Hz, 13.43 Hz, and 15.87 Hz, with sources redistributed to Brodmann areas 6, 4, 5, 3, and 31 (Figure 4B). These changes reflect a neurophysiological shift toward improved sensorimotor integration and emotional regulation. sLORETA (Figure 4C) showed normalized Z-scores (<1.5) in previously hyperactive areas and redistribution toward midline frontal structures (BA 24, 31).

This case exemplifies how REAC BWO-G may support the rebalancing of neurophysiological patterns involved in attentional and emotional regulation under exposure to chronic stress. The patient’s clinical improvements, better sleep, reduced headaches and facial flushing, and enhanced mood stability align with the observed cortical reorganization and increased self-regulatory capacity.

Case 3

A 36-year-old female with chronic dermographic urticaria, skin hypersensitivity, anxiety, anticipatory fears, somatic hypervigilance characterized by an exaggerated awareness and amplification of bodily sensations, myalgias presenting as diffuse and recurrent muscle pain without a specific organic cause, and persistent sleep difficulties, including trouble initiating and maintaining sleep, described a long-standing pattern of stress-related dysregulation. She had previously attempted dietary modifications, topical therapies, antihistamines, and relaxation exercises without sustained benefit.

**Figure 5:**
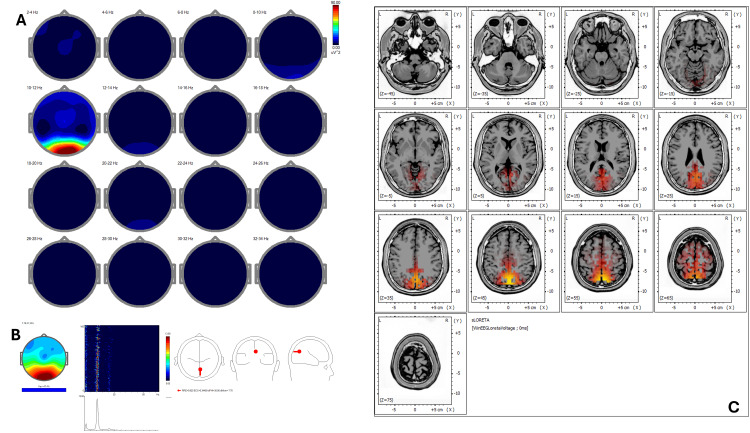
Case 3 - pre-treatment (A) EEG power spectral maps before REAC BWO-G treatment. Topographical EEG power maps in eyes-closed resting state show increased delta and alpha power over posterior and parietal regions, with reduced low-beta activity. This pattern is indicative of a reactive cortical state associated with somatic hypervigilance and stress-related dysregulation. (B) ICA before REAC BWO-G treatment. ICA decomposition reveals dominant peaks at 1.71 Hz, 10.01 Hz, and 20.75 Hz, with minor spectral activity around 11.47 Hz is not clearly distinguishable in the figure, with cortical sources localized to Brodmann areas 7, 19, 31, 18, and 5. These regions are implicated in visual processing, sensorimotor integration, and self-referential cognition, reflecting a state of heightened environmental reactivity. (C) sLORETA cortical source localization before REAC BWO-G treatment. sLORETA analysis shows hyperactivity (Z-score >2.5) in the posterior cingulate cortex (BA 31) and visual association areas (BA 18 and 19), consistent with stress-induced somatic hypervigilance and emotional dysregulation EEG: electroencephalography; ICA: Independent Component Analysis; REAC BWO-G: Radio Electric Asymmetric Conveyer Brain Wave Optimization Gamma; sLORETA: standardized low-resolution electromagnetic tomography

Symptoms negatively impacted her work performance and interpersonal relationships, reinforcing a maladaptive stress response. Baseline qEEG (Figure 5A) showed increased delta and alpha power over posterior and parietal regions with low-beta desynchronization. This electrophysiological pattern was consistent with reduced cortical efficiency, heightened sensory reactivity, and altered integration of somatosensory and visual inputs, changes that aligned with the patient’s somatic hypervigilance, hypersensitivity, and persistent muscle tension. ICA (Figure 5B) revealed peaks at 1.71, 10.01, 11.47, and 20.75 Hz, with sources in BA 7, 19, 31, 18, and 5, areas implicated in visual processing, sensorimotor integration, and self-referential cognition. sLORETA (Figure 5C) showed hyperactivity (Z-score >2.5) in the posterior cingulate (BA 31) and visual association areas, further supporting the link between abnormal network activation and the patient’s heightened sensory awareness and emotional dysregulation.

**Figure 6:**
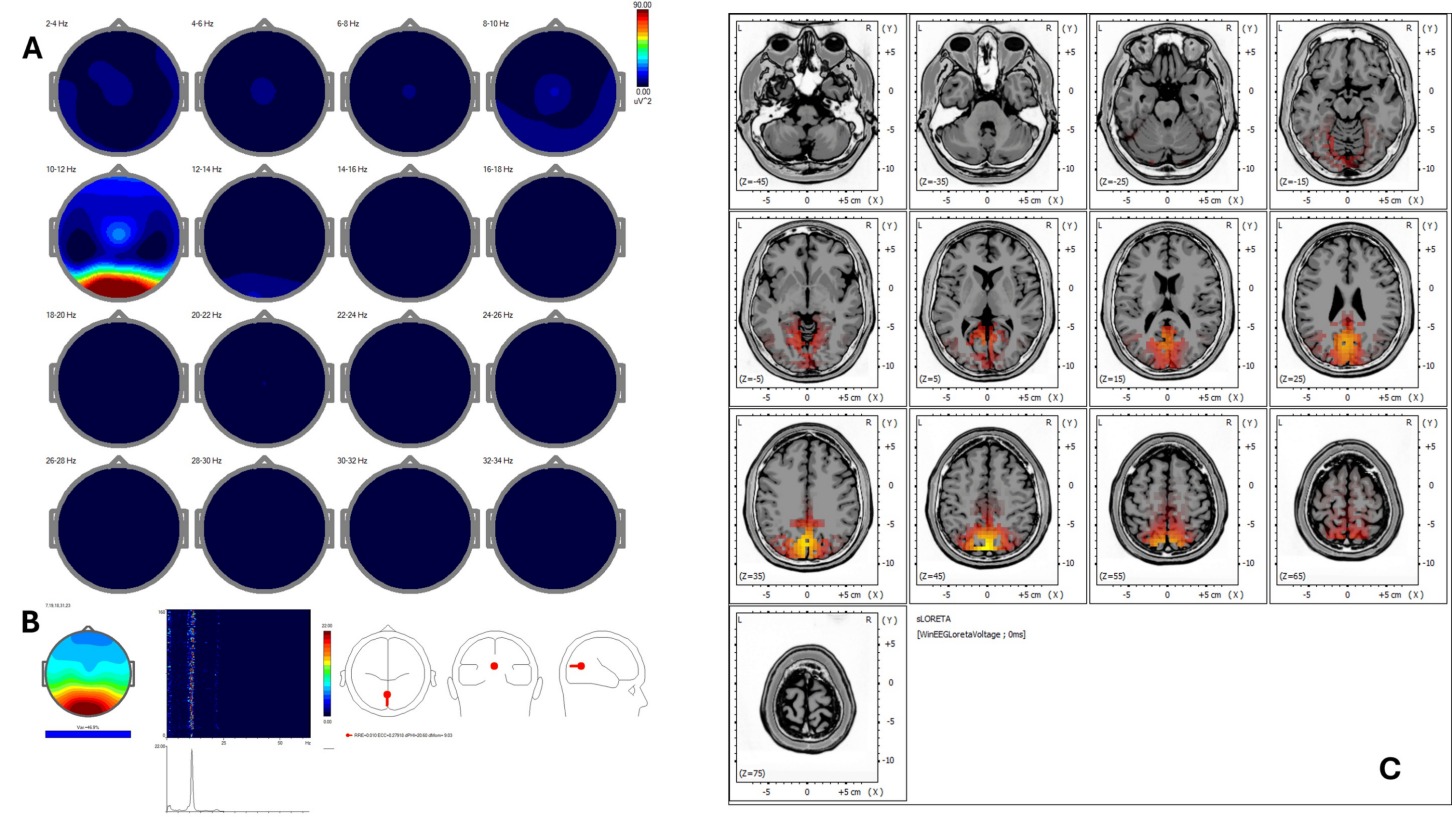
Case 3 - post-treatment (A) EEG power spectral maps after REAC BWO-G treatment. Post-treatment qEEG maps demonstrate improved power symmetry, reduction of delta and alpha hyperactivity, and increased low-beta synchronization, indicating a shift toward a more regulated and stable neurophysiological state. (B) ICA after REAC BWO-G treatment. ICA post-treatment reveals reorganized peaks at 1.71 Hz and 10.50 Hz, with sources redistributed to Brodmann areas 7, 19, 18, 31, and 23. This pattern reflects enhanced integration of sensory, cognitive, and emotional networks following neuromodulation. (C) sLORETA cortical source localization after REAC BWO-G treatment. sLORETA post-treatment analysis shows normalized Z-scores (<1.5) and a redistribution of activation toward the retrosplenial cortex and posterior midline structures, consistent with improved self-referential processing and emotional regulation EEG: electroencephalography; ICA: Independent Component Analysis; REAC BWO-G: Radio Electric Asymmetric Conveyer Brain Wave Optimization Gamma; sLORETA: standardized low-resolution electromagnetic tomography

Post-treatment qEEG (Figure 6A) demonstrated increased power symmetry and reduced low-frequency hyperactivity. ICA (Figure 6B) showed peaks at 1.71, 10.50, and 11.96 Hz, with sources redistributed to BA 7, 19, 18, 31, and 23. sLORETA (Figure 6C) showed normalization (Z-score <1.5) and increased engagement of the retrosplenial cortex.

This case demonstrates how REAC BWO-G may facilitate neurofunctional reorganization in limbic and sensory networks involved in emotional and somatic stress reactivity. The patient’s improved sleep, emotional stability, and reduction in urticaria and flushing episodes suggest enhanced neurophysiological resilience.

Case 4

A 27-year-old female, employed in the same occupational environment, presented with a complex pattern of dysregulation, clinically compatible with generalized anxiety disorder (GAD) but without a formal psychiatric diagnosis, including persistent cognitive disorganization, episodes of mental confusion, emotional lability, non-restorative sleep, fatigue, and difficulty maintaining attention and task focus. She reported frequent sensations of sensory overload, leading to heightened irritability and emotional reactivity, particularly in response to work-related stressors. Her professional activities required multitasking, problem-solving, and interpersonal interactions, which she described as exacerbating her sense of cognitive fragmentation and emotional distress. Previous pharmacological treatments with anxiolytics were discontinued due to side effects, and non-pharmacological interventions, such as dietary changes, mindfulness practices, and relaxation techniques, had been ineffective.

**Figure 7:**
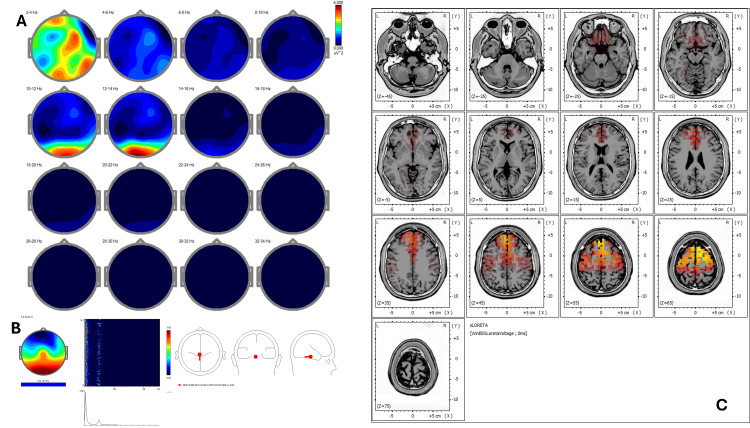
Case 4 - pre-treatment (A) EEG power spectral maps before REAC BWO-G treatment. Topographical EEG power maps (2–34 Hz) in eyes-closed resting state reveal elevated delta and alpha power in frontal and parietal regions, consistent with cortical slowing, somatic hypervigilance, and stress-induced dysregulation. (B) ICA before REAC BWO-G treatment. ICA decomposition highlights frequency peaks at 1.22 Hz and 11.96 Hz, with source localization in Brodmann areas 6, 4, 5, 3, and 31. This pattern reflects altered sensorimotor processing and emotional regulation under chronic stress. (C). sLORETA cortical source localization before REAC BWO-G treatment. sLORETA shows hyperactivity (Z-score >2.5) in the posterior cingulate cortex (BA 31) and sensorimotor areas (BA 6, 4), indicating dysregulated self-referential processing and autonomic imbalance EEG: electroencephalography; ICA: Independent Component Analysis; REAC BWO-G: Radio Electric Asymmetric Conveyer Brain Wave Optimization Gamma; sLORETA: standardized low-resolution electromagnetic tomography

Baseline qEEG (Figure 7A) revealed diffuse low-beta desynchronization and increased delta/theta activity in frontal-midline regions, suggesting impaired regulatory control. ICA (Figure 7B) identified peaks at 0.98, 2.20, 11.23, and 13.18 Hz, with sources in BA 6, 8, 32, 24, and 31, consistent with hyperactivation in medial prefrontal and cingulate regions involved in emotional processing and self-referential cognition. sLORETA (Figure 7C) showed hyperactivity (Z-score >2.5) in the anterior cingulate cortex (BA 32) and medial prefrontal cortex (BA 10), corroborating the clinical impression of emotional flooding, attentional dysregulation, and impaired top-down modulation of emotional responses.

**Figure 8:**
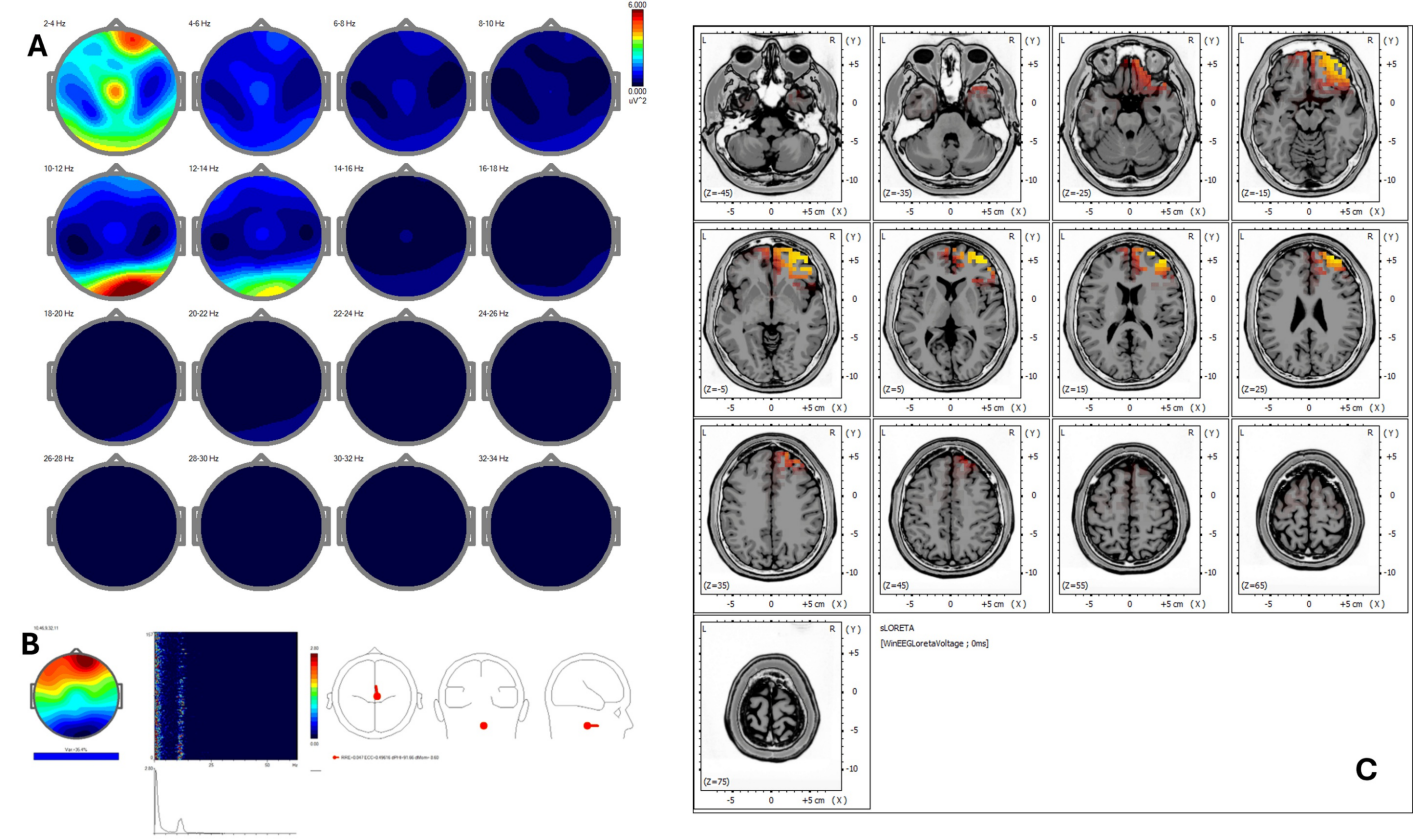
Case 4 - post-treatment (A) EEG power spectral maps after REAC BWO-G treatment. Post-treatment EEG maps show reduced delta and alpha power, improved spatial symmetry, and increased beta activity, suggesting restored cortical regulation and reduced hypervigilance. (B) ICA after REAC BWO-G treatment. Post-treatment ICA reveals reorganized peaks at 1.22 Hz and 10.50 Hz, with source redistribution toward Brodmann areas 7, 18, 19, and 23, reflecting improved integration of sensory, cognitive, and emotional networks. (C) sLORETA cortical source localization after REAC BWO-G treatment. sLORETA demonstrates normalization of previously hyperactive regions (Z-score <1.5) and a shift of cortical activation toward midline structures (BA 31 and 23), consistent with enhanced self-regulatory capacity and reduced stress-related dysregulation EEG: electroencephalography; ICA: Independent Component Analysis; REAC BWO-G: Radio Electric Asymmetric Conveyer Brain Wave Optimization Gamma; sLORETA: standardized low-resolution electromagnetic tomography

Following the 18-session REAC BWO-G cycle, post-treatment qEEG (Figure 8A) demonstrated improved frequency coherence, reduced asymmetry, and a more balanced power distribution across cortical regions. ICA (Figure 8B) showed reorganized peaks at 1.95, 2.20, 10.74, and 12.70 Hz, with source redistribution to BA 10, 46, 9, 32, and 11, corresponding to enhanced engagement of executive control and emotion regulation networks. Post-treatment sLORETA (Figure 8C) demonstrated normalization (Z-score <1.5) in previously hyperactive regions and increased engagement of midline cortical structures, including the dorsal anterior cingulate and medial prefrontal areas, suggesting improved integration of emotional and cognitive processing.

This case illustrates the capacity of REAC BWO-G to rebalance prefrontal and cingulate cortical activity associated with stress-related anxiety and executive dysfunction. The patient's improvements in mood stability, concentration, and coping occurred without any external changes, supporting the neuromodulatory impact of the intervention.

Case 5

A 33-year-old male with a diagnosis of ADHD established during adolescence, and currently not receiving pharmacological treatment due to discontinuation of stimulant medications for side effects, presented with stress vulnerability, attention deficits, impulsivity, emotional lability, persistent musculoskeletal tension and non-specific pain symptoms, and fatigue, describing a longstanding pattern of stress-related maladaptation. He worked in a high-demand technical environment, where performance pressure, multitasking, and constant problem-solving were required, and he reported these demands as exacerbating his ADHD symptoms. Previous stimulant treatments provided partial relief but were discontinued due to side effects. He sought non-pharmacological intervention for improved focus and emotional regulation.

**Figure 9:**
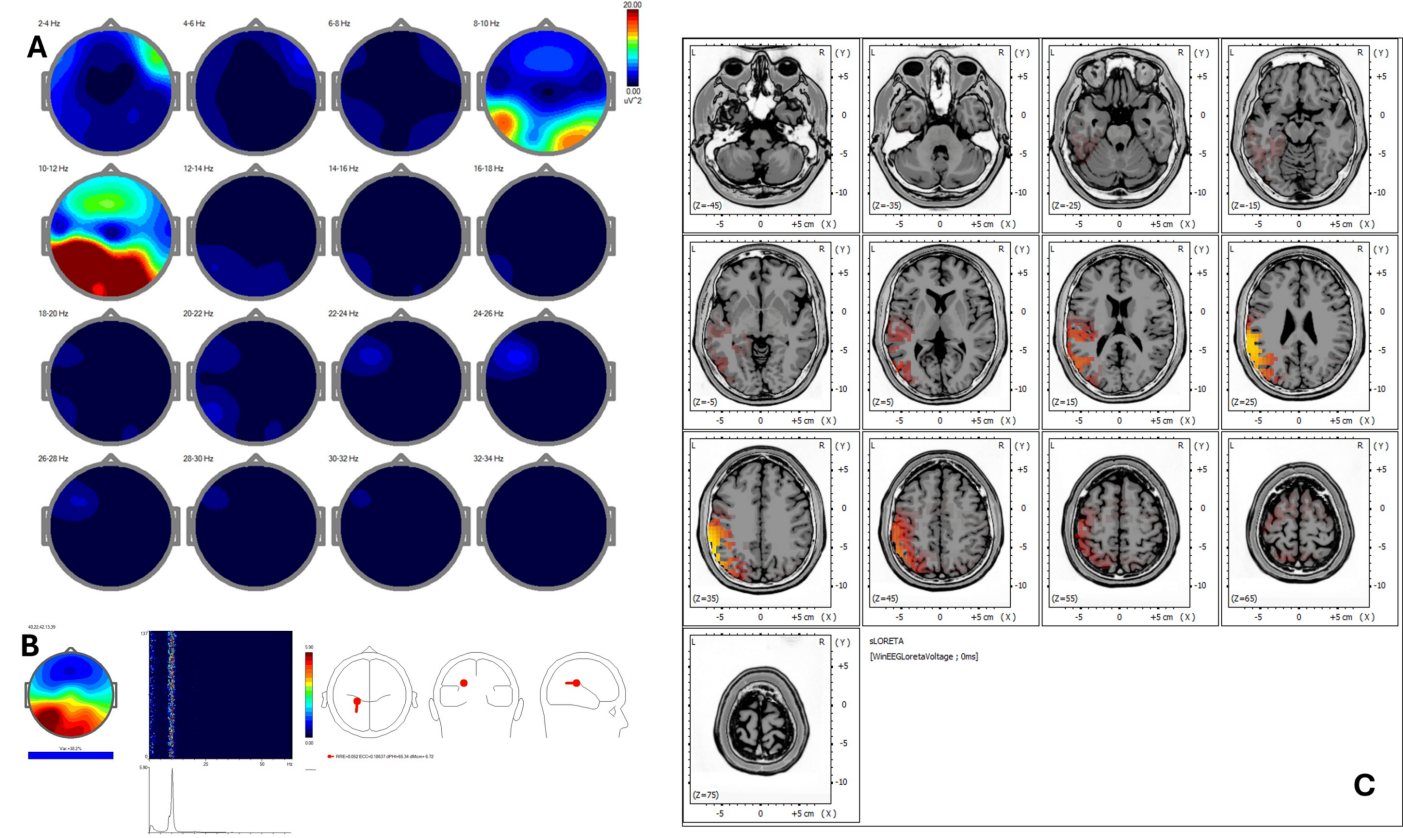
Case 5 - pre-treatment (A) EEG power spectral maps before REAC BWO-G treatment. Topographical EEG power maps (2-34 Hz) in eyes-closed resting state show reduced beta coherence and increased delta/theta power predominantly over posterior regions, suggesting a pattern of cortical slowing and stress-related dysregulation, particularly in attentional and sensory processing networks. (B) ICA before REAC BWO-G treatment. ICA decomposition reveals peaks at 1.22 Hz, 9.03 Hz, and 10.74 Hz, with sources localized to Brodmann areas 40, 22, 42, 13, and 39. These regions are associated with language, somatosensory integration, and attentional processing, reflecting a neurophysiological pattern of impaired executive control and sensory hyperreactivity. (C) sLORETA cortical source localization before REAC BWO-G treatment. sLORETA analysis demonstrates hyperactivity (Z-score >2.5) in the temporoparietal junction (BA 39) and insular cortex (BA 13), regions implicated in sensory integration, interoception, and emotional awareness, aligning with the patient's ADHD symptoms and stress-related maladaptation EEG: electroencephalography; ICA: Independent Component Analysis; REAC BWO-G: Radio Electric Asymmetric Conveyer Brain Wave Optimization Gamma; sLORETA: standardized low-resolution electromagnetic tomography

Baseline qEEG (Figure 9A) revealed reduced beta coherence and increased delta/theta over posterior regions. ICA (Figure 9B) showed peaks at 1.22, 9.03, and 10.74 Hz, with sources in BA 40, 22, 42, 13, and 39. sLORETA (Figure 9C) highlighted hyperactivity (Z-score >2.5) in the temporoparietal junction (BA 39) and insular cortex (BA 13).

**Figure 10:**
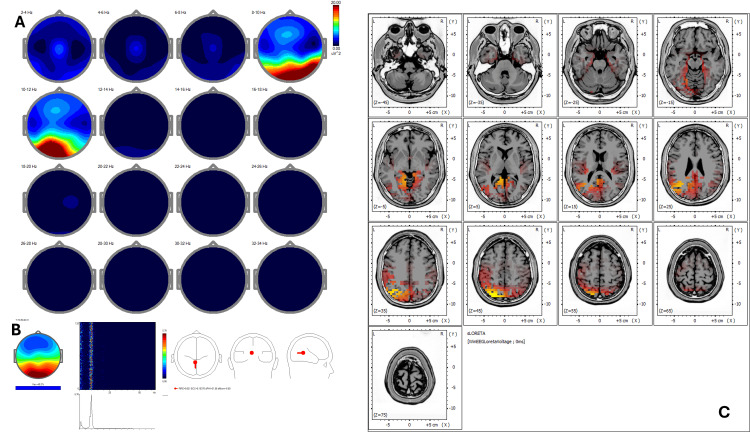
Case 5 - post-treatment (A) EEG power spectral maps after REAC BWO-G treatment. Post-treatment EEG maps reveal improved beta synchronization, reduced low-frequency dominance, and enhanced spatial symmetry, suggesting improved cortical regulation and functional connectivity. (B) ICA after REAC BWO-G treatment. Post-treatment ICA shows reorganized peaks at 1.46 Hz, 9.77 Hz, and 10.99 Hz, with source redistribution to Brodmann areas 7, 19, 39, 40, and 31. This reorganization reflects a neurophysiological shift toward improved attentional control, sensory processing, and emotional regulation. (C). sLORETA cortical source localization after REAC BWO-G treatment. sLORETA post-treatment reveals reduced hyperactivity (Z-score <1.5) in previously affected areas, with normalized activity in the temporoparietal and insular cortices, indicating a rebalancing of networks implicated in attention, emotional regulation, and interoception EEG: electroencephalography; ICA: Independent Component Analysis; REAC BWO-G: Radio Electric Asymmetric Conveyer Brain Wave Optimization Gamma; sLORETA: standardized low-resolution electromagnetic tomography

Post-treatment qEEG (Figure 10A) demonstrated improved beta synchronization and reduced low-frequency dominance. ICA (Figure 10B) revealed peaks at 1.46, 9.77, and 10.99 Hz, with sources redistributed to BA 7, 19, 39, 40, and 31. sLORETA (Figure 10C) showed reduced hyperactivity (Z-score <1.5) in previously affected areas.

This case highlights the potential of REAC BWO-G to promote neurofunctional reorganization in networks involved in attention and emotional regulation. The patient had a formal diagnosis of ADHD established during adolescence. The qualifier ‘stress-related’ reflects the clinical observation that his attentional deficits, impulsivity, and emotional lability were consistently exacerbated by prolonged exposure to high-demand occupational settings and performance pressure. The observed clinical improvements in focus, emotional control, and sleep following REAC BWO-G suggest enhanced self-regulation in this patient, particularly in managing symptoms aggravated by chronic stress exposure.

All five participants reported improvements in emotional regulation, cognitive clarity, and sleep quality. Although these findings are based on self-reports and no validated psychometric assessments were administered in this retrospective case series, all participants underwent clinical evaluation before and after the treatment cycle by expert medical personnel, who corroborated the reported changes. These observations align with previous results from studies using REAC neuromodulation protocols (e.g., NPPO and NPPO-CB), where validated psychometric instruments demonstrated significant clinical benefits.

Following the detailed presentation of each case, a comparative summary is provided to consolidate the key findings across all patients (Table 1). This table serves to illustrate the neurophysiological changes observed pre- and post-treatment, as well as the associated clinical outcomes. The inclusion of this data facilitates an integrated understanding of the consistent patterns emerging from this case series and underscores the potential of REAC BWO-G in modulating dysfunctional cortical dynamics associated with chronic stress (Table 1).

**Table 1:** Neurophysiological changes before and after REAC BWO-G treatment The symbol “**” indicates that Z-score thresholds >2.5 and <1.5 were used to define, respectively, pre-treatment cortical hyperactivity and post-treatment normalization in the sLORETA analyses. Clinical outcomes were evaluated through patient self-reports, corroborated by pre- and post-treatment clinical examinations conducted by expert medical personnel ↑: increase; ↓: decrease; BA: Brodmann area; qEEG: quantitative electroencephalography; ICA: Independent Component Analysis; REAC BWO-G: Radio Electric Asymmetric Conveyer Brain Wave Optimization Gamma; sLORETA: standardized low-resolution electromagnetic tomography

Case	Age, years	Sex	qEEG findings (Pre)	qEEG findings (Post)	ICA peaks (Hz) (pre)	ICA peaks (Hz) (post)	sLORETA findings (pre Z >2.5)^**^	sLORETA findings (post Z <1.5)^**^	Clinical outcome at 1 month
1	61	F	Delta/Alpha ↑ (BA 4,5,6,7,31)	Symmetry ↑ (BA 23,24,30,31,7)	0.98, 1.95, 8.79, 10.25, 12.70, 21.23	9.28, 10.25, 12.70, 24.17	Hyperactivity BA 4,6,7,31	Normalization BA 30,31	Sleep ↑, mood ↑, tension ↓
2	35	M	Delta/Alpha ↑ (BA 39,22,19,40,13)	Symmetry ↑ (BA 6,4,5,3,31)	1.22, 10.99, 14.89, 17.33	1.71, 11.47, 13.43, 15.87	Hyperactivity BA 39,22	Normalization BA 6,4,31	Sleep ↑, headaches ↓, mood ↑
3	36	F	Delta/Alpha ↑ (BA 7,19,31,18,5)	Symmetry ↑ (BA 7,19,18,31,23)	1.71, 10.01, 11.47, 20.75	1.71, 10.50, 11.96	Hyperactivity BA 31,7,19	Normalization BA 23,31	Urticaria ↓, sleep ↑, mood ↑
4	27	F	Delta/Theta ↑ (BA 6,8,32,24,31)	Coherence ↑ (BA 10,46,9,32,11)	0.98, 2.20, 11.23, 13.18	1.95, 2.20, 10.74, 12.70	Hyperactivity BA 32,10	Normalization BA 10,32	Anxiety ↓, cognition ↑, mood ↑
5	33	M	Delta/Theta ↑ (BA 40,22,42,13,39)	Beta ↑ (BA 7,19,39,40,31)	1.22, 9.03, 10.74	1.46, 9.77, 10.99	Hyperactivity BA 39,13	Normalization BA 31,7,40	Attention ↑, impulsivity ↓, mood ↑

All patients were clinically followed up for at least one month after completing the treatment cycle, during which no adverse effects were reported and clinical improvements were maintained. While no further follow-up was performed in this retrospective analysis, future prospective studies are planned to assess the long-term stability and reproducibility of these effects.

## Discussion

This case series provides an exploration of the neurophysiological effects of REAC BWO-G neuromodulation in individuals with chronic stress-related dysregulation. The consistent trends observed across cases, including the reduction of delta and theta hyperactivity, increased beta coherence, and the reorganization of cortical activity toward midline structures (such as the posterior cingulate and retrosplenial cortex), align with existing literature on stress-related alterations in brain dynamics [28]. Studies have demonstrated that chronic stress is associated with increased low-frequency EEG activity (delta and theta), decreased beta coherence, and functional disruptions within the DMN, SN, and CEN [29,30]. For example, reduced DMN connectivity has been linked to maladaptive rumination and impaired emotional self-regulation [31], while SN hyperactivity has been associated with heightened threat perception and somatic hypervigilance [32]. Likewise, CEN hypoconnectivity correlates with deficits in working memory and cognitive control, limiting adaptive responses to stress [30].

These neurophysiological alterations contribute to the persistence of dysregulated emotional and autonomic states in individuals exposed to prolonged psychosocial stress. The neurophysiological changes observed in this series correspond with improvements reported by patients in sleep quality, mood stability, and cognitive clarity. These effects are thought to arise from the interaction between asymmetrically conveyed radioelectric fields and areas of altered endogenous bioelectrical activity in target tissues. This interaction promotes the progressive optimization of bioelectrical activity disrupted by epigenetic processes, fostering the recovery of physiological functional patterns. Bioelectrical modulation has been shown to influence cellular signaling, gene expression, and functional connectivity, counteracting maladaptive neuroplastic changes induced by chronic stress or other adverse conditions [19,33].

As previously demonstrated in multiple studies on REAC neuromodulation protocols, this modulation supports the rebalancing of endogenous brain rhythms [34], with specific evidence for the restoration or functional optimization of gamma band activity, which plays a pivotal role in cognitive integration, working memory, and emotional regulation [35,36]. For example, Uhlhaas and Singer [35] demonstrated that gamma oscillations are critical for perceptual binding and higher-order cognitive processes, while disruptions in gamma synchronization have been linked to impairments in attention, memory, and affective regulation. Clinical data from REAC BWO-G case studies have shown concurrent improvements in gamma rhythm organization and behavioral outcomes, including enhanced emotional stability and cognitive flexibility [20,21,27]. This understanding reinforces the conceptual foundation of REAC BWO-G as a targeted intervention to promote neurophysiological resilience and emotional well-being in individuals affected by chronic stress.

While the convergence of neurophysiological and clinical findings suggests a specific modulatory effect of REAC BWO-G, it is important to acknowledge the inherent limitations of this case series. The absence of a control group, the small sample size, and the lack of blinded EEG analysis constrain the ability to quantify effect size and confirm causality with statistical rigor. Another limitation concerns the timing of EEG recordings. These were generally performed in the morning (between 9:00 and 12:00), but timing was not strictly standardized. Since neurophysiological parameters and clinical outcomes such as cognition, sleep, and emotional balance are influenced by circadian rhythms, this factor should be considered when interpreting the results. Future studies should aim to control for time-of-day effects and investigate the role of individual chronotypes.

Furthermore, although all participants reported improved emotional regulation, sleep, and cognitive clarity, this retrospective case series did not employ standardized psychometric tools. Clinical improvements were evaluated through structured interviews conducted by expert medical personnel, who assessed symptom changes based on patient self-reports, clinical observation, and corroborating feedback from family members or colleagues. These qualitative findings were correlated with objective neurophysiological changes observed in qEEG, ICA, and sLORETA analyses, providing convergent evidence for the reported functional improvements. Future research should incorporate such measures to enable quantification and better alignment between subjective and objective outcomes. Sex and age may also influence baseline neurophysiology and responsiveness to treatment. While this study was not designed to detect subgroup effects, future investigations should explore these variables using stratified analyses. While this case series was not powered to detect subgroup effects, future research should explore these demographic variables through stratified analyses.

However, it should be noted that the long-term stability of bioelectrical modulation, including the restoration of functional symmetry [34,37], has already been demonstrated in previous clinical studies. In the present case series, patients were clinically followed for at least one month after completing the treatment cycle, during which no adverse effects or symptom relapses were observed. Furthermore, it is important to clarify that the BWO-G treatment is not intended as a single, definitive intervention. While significant individual improvements were observed, it remains possible that future environmental stressors may re-induce bioelectrical dysregulation, necessitating additional treatment cycles as part of a periodic maintenance strategy. This consideration aligns with the broader therapeutic concept of maintaining neuro-psycho-physical resilience and adaptive coping abilities over time.

The clinical significance of the observed neurophysiological changes is supported by prior research indicating that increased beta coherence and the engagement of midline cortical structures such as the posterior cingulate cortex are associated with improved executive function, enhanced emotional regulation, and reduced hyperarousal states. For instance, increased frontal beta coherence has been linked to better cognitive control and working memory performance in healthy adults [38]. The posterior cingulate cortex, a key node in self-referential and attentional networks, has been proposed to regulate arousal and internally focused cognition [39]. Additionally, enhanced beta synchronization within fronto-parietal circuits may reflect compensatory mechanisms in conditions such as depression, supporting memory and attention retention [40]. Effective connectivity between medial frontal and posterior cingulate regions has been shown to correlate with working memory efficiency in psychiatric populations [41]. These findings suggest that REAC BWO-G may facilitate the restoration of adaptive network dynamics underlying stress resilience.

Compared to other noninvasive neuromodulation techniques such as TMS and tDCS, REAC BWO-G offers a distinct approach based on the interaction with endogenous bioelectrical activity rather than the delivery of external currents or magnetic fields. The simplicity of the protocol, the absence of operator-dependent parameter adjustments, the standardized and non-modifiable emission parameters, and the absence of reported adverse effects further highlight the potential clinical feasibility and safety of this intervention. No adverse effects were reported during or after the treatment sessions, supporting the safety and tolerability of REAC BWO-G in this population.

These findings contribute to a paradigm shift in neuromodulation approaches, emphasizing the modulation of endogenous bioelectrical activity as a novel and promising therapeutic strategy. An important aspect of this approach is its favorable safety profile. REAC protocols, including BWO-G, have been extensively applied in diverse clinical populations for many years without reports of harmful biological or genetic effects. The bioelectrical modulation induced by REAC technology targets only areas where endogenous bioelectrical activity is altered, progressively restoring physiological patterns without forcing activity in unaffected regions. Given this mechanism, there is no theoretical or experimental evidence suggesting risks of unwanted genetic modifications or adverse biological consequences, even in long-term follow-up from previous studies [37,42]. They also suggest practical implications for clinicians and healthcare systems, where REAC BWO-G could be considered as an accessible, noninvasive therapeutic option in stress-related disorders.

The lack of a control group, such as healthy individuals or a placebo condition, is a recognized limitation of this case series design. Nevertheless, the goal of this work was to document consistent clinical and neurophysiological changes across individuals subjected to the same REAC BWO-G protocol. Future research should include larger and more heterogeneous cohorts, standardized psychometric and neurophysiological assessments, and extended follow-up to confirm the persistence of benefits. Comparative trials with other neuromodulation approaches, as well as studies combining REAC BWO-G with behavioral or rehabilitative interventions, may further optimize treatment outcomes in stress-related and neurocognitive disorders.

## Conclusions

This case series underscores the potential of REAC BWO-G as a noninvasive neuromodulation strategy for promoting functional brain reorganization in chronic stress-related symptoms and conditions. The integration of qEEG, ICA, and sLORETA analyses provides a robust framework for assessing treatment effects and reveals consistent neurophysiological changes across multiple cases. The observed reductions in delta and theta activity and the increases in alpha, beta, and gamma power are in line with prior research showing that these neurophysiological patterns are associated with improved emotional regulation, enhanced cognitive flexibility, and better autonomic balance in stress-related conditions. The redistribution of cortical sources toward midline structures, particularly the posterior cingulate cortex and related areas, reflects a shift toward more integrated and adaptive network dynamics, as previously linked to improved executive function and stress resilience. These findings, in line with previous case reports, highlight the reproducibility of REAC BWO-G’s effects and endorse its potential as a repeatable, safe, and accessible intervention for stress-related dysregulation. Further studies with larger samples, control groups, and extended follow-up are warranted to confirm these results and explore the broader applicability of REAC BWO-G in clinical practice, including its potential role in addressing neuropsychiatric and neurodevelopmental disorders due to chronic stress exposure and related conditions and dysfunctional cortical activity. The observed neurophysiological changes and clinical improvements are consistent with the progressive recovery of endogenous gamma activity promoted by the REAC BWO-G protocol, as part of the broader family of NPPO-CB treatments.
